# Supplementary material for: Synergistic Inhibitory Effect of Multiple Polyphenols from Spice on Acrolein during High-Temperature Processing
Source: Foods. 2023 Jun 9;12(12):2326. doi: 10.3390/foods12122326 (PMC10296921; doi:10.3390/foods12122326)
Supplement: Supplementary file 1 [file foods-12-02326-s001.zip › Table S1.pdf]

Table S1 Sensory characteristics of roasted pork.

Indicator	group A	group B	group C	group D	group E	group F
Appearance	4.92 ± 0.06^{a}	4.81 ± 0.04^{c}	4.87 ± 0.08^{b}	4.76 ± 0.06^{d}	4.61 ± 0.05^{f}	4.69 ± 0.07^{e}
Flavor and aroma	4.95 ± 0.04^{a}	4.92 ± 0.07^{b}	4.90 ± 0.08^{bc}	4.91 ± 0.06^{b}	4.87 ± 0.07^{d}	4.88 ± 0.05^{cd}
Tenderness	4.93 ± 0.06^{a}	4.88 ± 0.05^{b}	4.91 ± 0.04^{a}	4.86 ± 0.08^{b}	4.75 ± 0.07^{d}	4.78 ± 0.06^{c}
Color	4.95 ± 0.04^{a}	4.89 ± 0.06^{b}	4.86 ± 0.07^{c}	4.79 ± 0.05^{d}	4.70 ± 0.06^{e}	4.71 ± 0.07^{e}
View on the cut	4.91 ± 0.08^{a}	4.85 ± 0.07^{cd}	4.86 ± 0.08^{bc}	4.88 ± 0.05^{b}	4.83 ± 0.07^{de}	4.80 ± 0.05^{f}
Dampness	4.90 ± 0.07^{a}	4.76 ± 0.05^{c}	4.85 ± 0.07^{b}	4.67 ± 0.04^{d}	4.64 ± 0.05^{e}	4.69 ± 0.07^{d}
Taste	4.96 ± 0.03^{a}	4.90 ± 0.06^{b}	4.88 ± 0.05^{b}	4.85 ± 0.04^{c}	4.84 ± 0.07^{c}	4.83 ± 0.08^{c}

Note: $^{a\text{-}f}$ different letters in the same row represent significant differences among different samples ($P < 0.05$). Group A: control, group B: with the complex of [CAR + ALP + PIN] according to the fixed proportion of the three compounds in 1.0% AKH, group C: with AKH (1.0%), group D: with CUR (0.1%), group E: with [CAR + ALP + PIN] (1.0% AKH) + CUR (0.1%), and group F: with AKH (1.0%) + CUR (0.1%).
