# Supplementary figures and images for: Synergistic Inhibitory Effect of Multiple Polyphenols from Spice on Acrolein during High-Temperature Processing
Source: Foods. 2023 Jun 9;12(12):2326. doi: 10.3390/foods12122326 (PMC10296921; doi:10.3390/foods12122326)

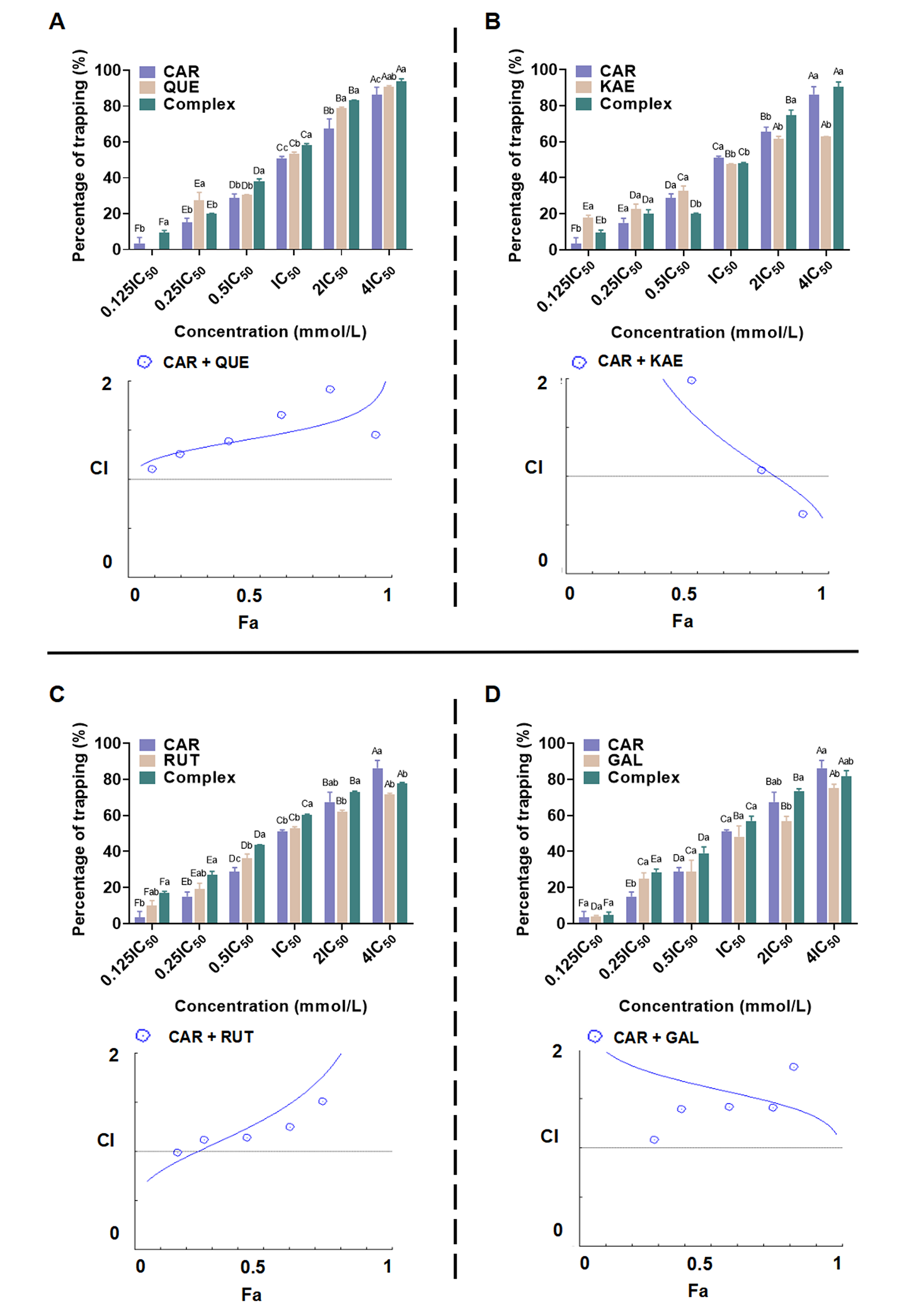

Supplement: Supplementary file 1 [file foods-12-02326-s001.zip › Fig. S1.tif]

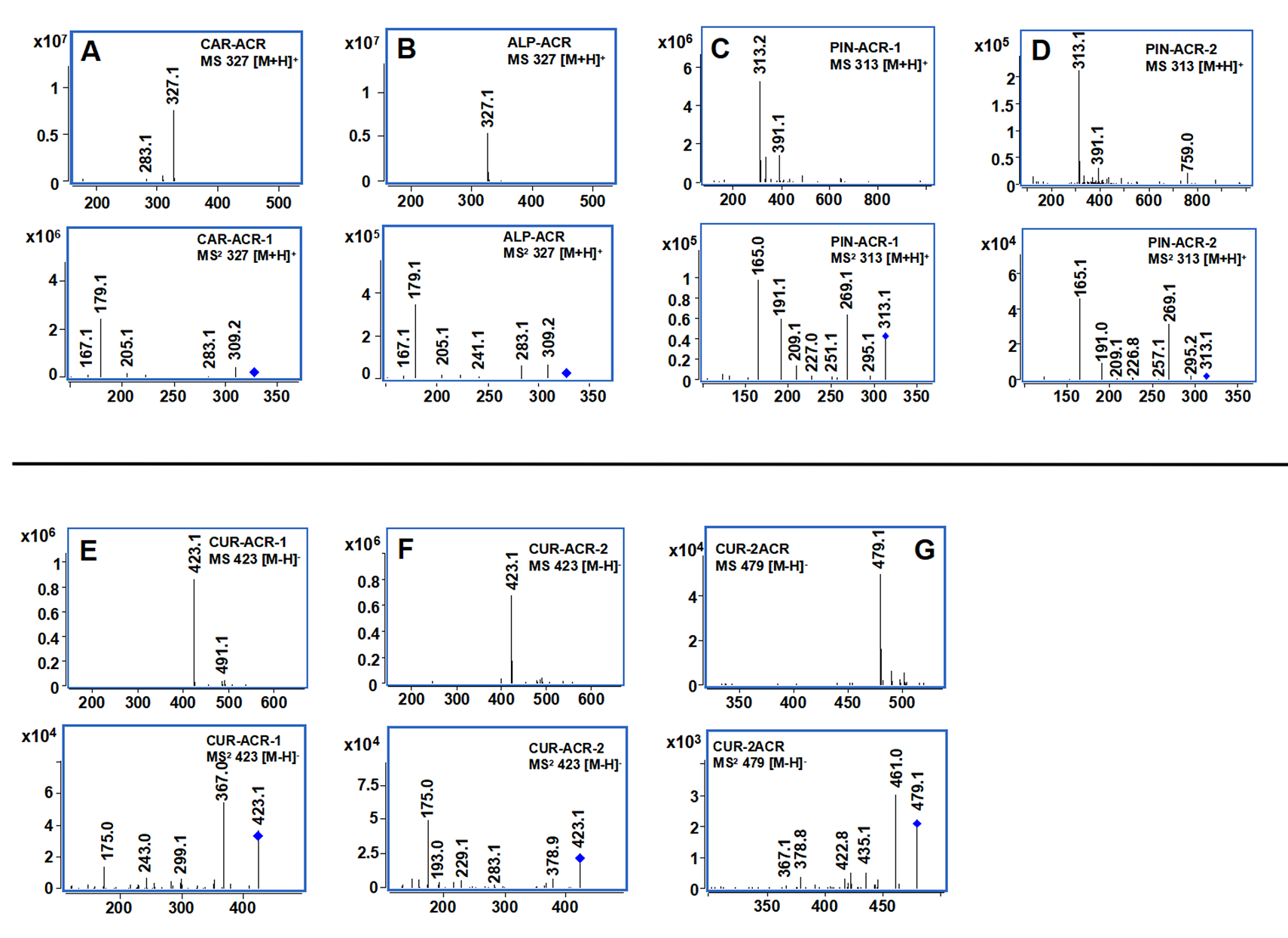

Supplement: Supplementary file 1 [file foods-12-02326-s001.zip › Fig. S2.tif]

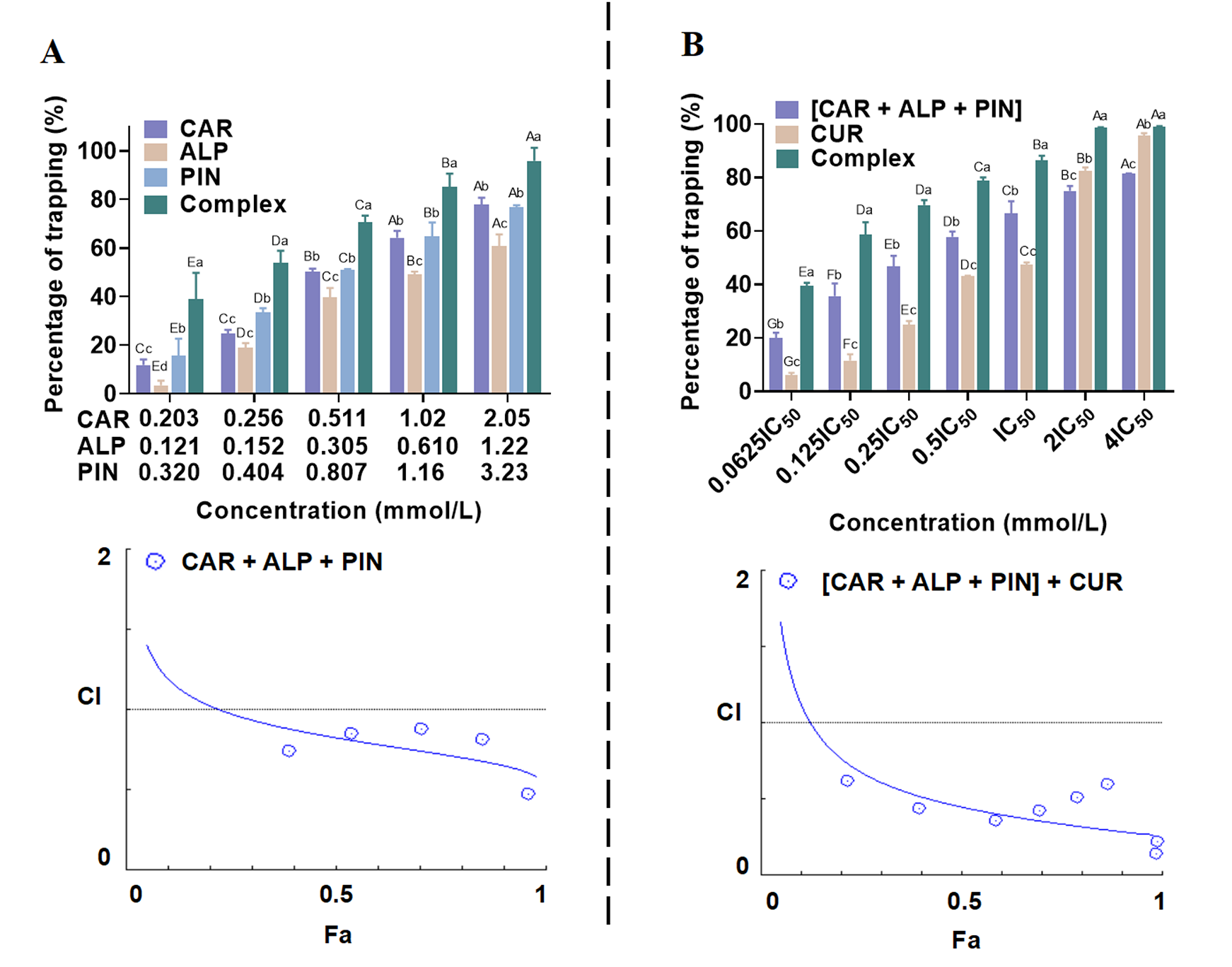

Supplement: Supplementary file 1 [file foods-12-02326-s001.zip › Fig. S3.tif]

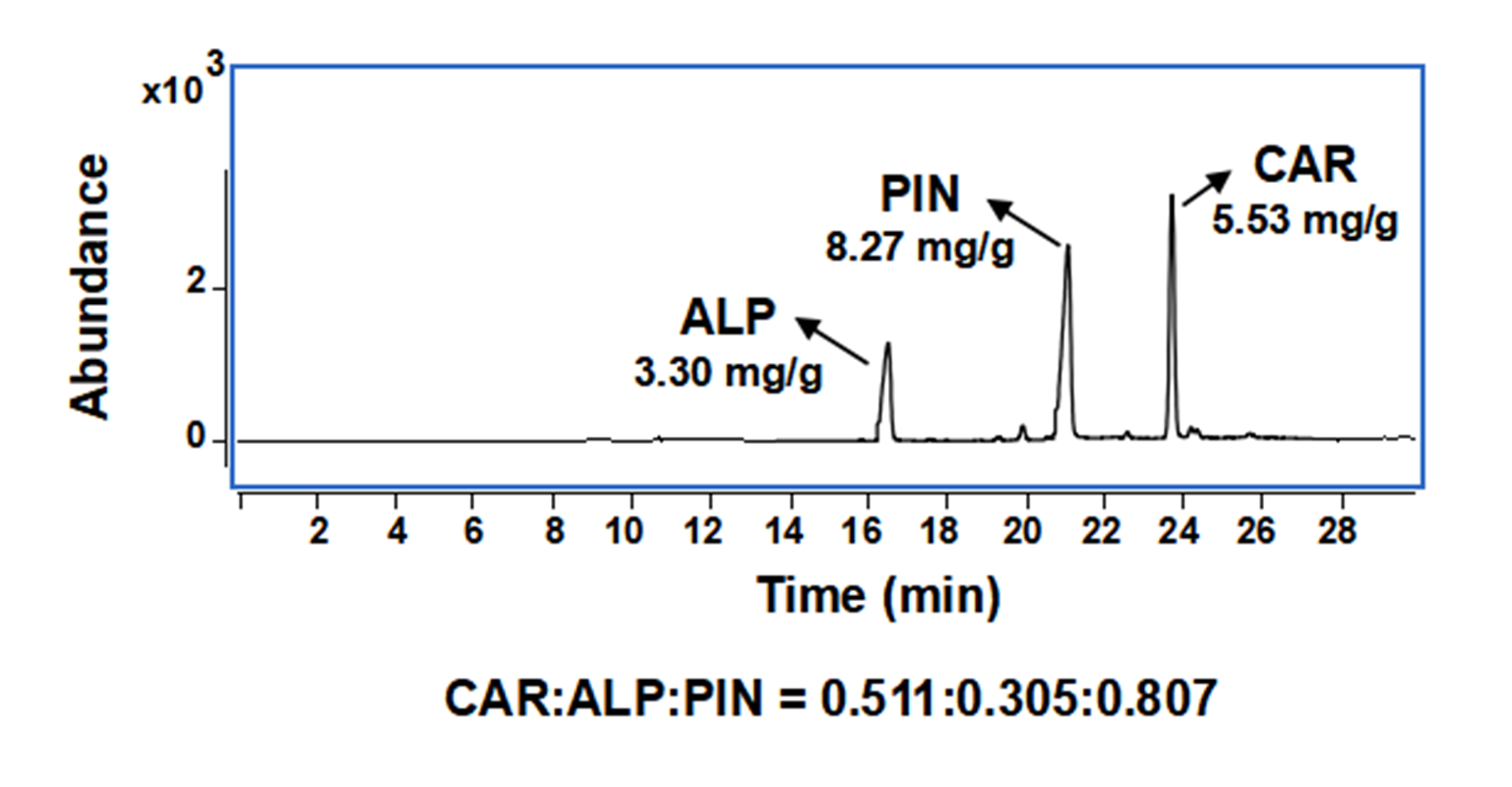

Supplement: Supplementary file 1 [file foods-12-02326-s001.zip › Fig. S4.tif]
